# Role of HIF1α and HIF2α in Cre Recombinase–Induced Retinal Pigment Epithelium Pathology and Its Secondary Effect on Choroidal Neovascularization

**DOI:** 10.1016/j.ajpath.2023.05.017

**Published:** 2023-06-16

**Authors:** Enrico Cristante, Sidath E. Liyanage, Alexander J. Smith, Robin R. Ali, James W.B. Bainbridge

**Affiliations:** ∗ UCL Institute of Ophthalmology London, United Kingdom; † Centre for Cell and Gene Therapy, King's College London, Guy's Hospital, London, United Kingdom; ‡ NIHR Biomedical Research Centre at Moorfields Eye Hospital NHS Foundation Trust, London, United Kingdom

## Abstract

*Cre*^*Trp1*^ mice are widely used for conditional retinal pigment epithelium (RPE) gene function studies. Like other *Cre/LoxP* models, phenotypes in *Cre*^*Trp1*^ mice can be affected by Cre-mediated cellular toxicity, leading to RPE dysfunction, altered morphology and atrophy, activation of innate immunity, and consequent impairment of photoreceptor function. These effects are common among the age-related alterations of RPE that feature in early/intermediate forms of age-related macular degeneration. This article characterizes Cre-mediated pathology in the *Cre*^*Trp1*^ line to elucidate the impact of RPE degeneration on both developmental and pathologic choroidal neovascularization. Nonredundant roles of the two major components of the hypoxia-inducible factor (HIF) family of transcription regulators, HIF1α and HIF2α, were identified. Genetic ablation of *Hif1a* protected against Cre-induced degeneration of RPE and choroid, whereas ablation of *Hif2a* exacerbated this degeneration. Furthermore, HIF1α deficiency protected *Cre*^*Trp1*^ mice against laser-induced choroidal neovascularization, whereas HIF2α deficiency exacerbated the phenotype. Cre-mediated degeneration of the RPE in *Cre*^*Trp1*^ mice offers an opportunity to investigate the impact of hypoxia signaling in the context of RPE degeneration. These findings indicate that HIF1α promotes Cre recombinase–mediated RPE degeneration and laser-induced choroidal neovascularization, whereas HIF2α is protective.

The retinal pigment epithelium (RPE) is a monolayer of pigmented neuroectoderm-derived cells on the Bruch basement membrane, between the choroidal capillary network and the neurosensory retina. The RPE constitutes the outer blood–retina barrier, which regulates the flux of nutrients, ions, and water from the choroidal capillary network to the neuronal layers of the inner retina. Other roles of the RPE include phagocytosis of shed photoreceptor outer segment discs, re-isomerization of the 11-*cis* retinal chromophore as part of the visual cycle, protection against photo-damage, and secretion of trophic factors.^1^^,^^2^ The RPE is essential for the ordered and timely development of the choroidal vascular network^3^ and neuroretina, and for maintaining their survival and function.^1^^,^^4^^,^^5^

Besides its role in retinal development, the RPE is centrally involved in the pathophysiology of a range of disabling ocular disorders.^2^^,^^6^ In particular, dysfunction of the pigment epithelium and the consequent disruption of its relationship with the choroidal vasculature are central to the development and progression of age-related macular degeneration (AMD).^7^

Given its central role in several retinal processes, the RPE has been extensively studied, both *in vitro* and *in vivo*, by employing tissue- and gene-specific targeting technologies such as the *Cre/LoxP* recombinase system.^8^ One of the most widely used murine transgenic lines is the *Cre*^*Trp1*^, which expresses Cre recombinase under the tyrosinase-related protein 1 gene (*Tyrp1*) promoter.^9^ This line was originally believed to target exclusively melanin-producing cells and tissues, such as the RPE and the iris. However, recent evidence of ectopic *Tyrp1* promoter–mediated Cre expression and activity in the neuroretina and optic nerve in the early postnatal period indicates developmental *Tyrp1* activity in late neuroprogenitor cells,^10^ which complicates the interpretation of ocular findings generated while using this mouse line for RPE-specific gene targeting. Thanos et al^11^ described a distinctive dose-dependent phenotype of RPE monolayer disorganization, cellular dysfunction and atrophy, localized recruitment of inflammatory cells, and activation and consequent impairment in photoreceptor activity in the *Cre*^*Trp1*^ line, mainly evident at the retinal periphery. These findings may have been attributable to the potent activity of Cre endonuclease, which can cause cellular toxicity by nonspecific DNA cleavage,^12^, ^13^, ^14^ and harmful genotoxic outcomes in the Cre-expressing tissue, especially in nonregenerative tissues such as the RPE.

The hypoxia-inducible transcription factors (HIFs) play a key role in generating transcriptional responses to physiological and pathologic stimuli. HIFs are involved in orchestrating ocular and retinal development and in mediating responses to hypoxia and inflammatory activation, processes that are closely implicated in AMD.^15^, ^16^, ^17^, ^18^, ^19^ Given these features, the authors sought to investigate the involvement of the HIF pathway in the aberrant phenotype of the *Cre*^*Trp1*^ line and in laser-induced choroidal neovascularization (CNV) in the context of Cre-mediated RPE pathology.

## Materials and Methods

### Animals

All *in vivo* procedures were conducted under the regulation of the UK Home Office Animals Act 1986. This work complied with The Association for Research in Vision and Ophthalmology's Statement for the Use of Animals in Ophthalmology and Vision Research. Both female and male littermates were included at similar numbers in each experimental group. The age of the animals, unless otherwise stated, was 6 to 12 weeks (young adult mice, from a neurodevelopmental perspective). The median age of all adult mice was 8 weeks.

The following lines were used (C57BL/6 background): transgenic mice expressing one copy of Cre recombinase under the control of Tyrp1, referred to as *Cre*^*Trp1*^,^9^^,^^20^ *Hif1a*^*f/f*^,^21^ *Hif2a*^*f/f*^,^22^ and combinations. RPE-specific homozygosity of Cre is known to lead to a severe degenerative phenotype as reported^11^; only one heterozygous *Cre*^*Trp1*^ male or female partner was utilized for breeding purposes to avoid homozygosity in littermates. Regular littermate genotyping for *Cre* expression was employed to confirm the absence of homozygosity in breeders. Some of these combinations were crossed with (*ROSA*)26 mT/mG^23^ to quantify Cre recombination efficiency. The group controls included all of the Cre-negative littermate genotypes (wild-type C57BL/6 and *Hif*x^*f/f*^ lines). All lines were kept on a standard 12/12-hour light/dark cycle with food and water *ad libitum*. Genotyping was performed as previously described,^20^ and all lines were confirmed as *rd8* (*Crb1*^*rd8/rd8*^) mutation free.^24^ For *in vivo* procedures, mice were anesthetized using i.p. injections of Dormitor (medetomidine, 1 mg/mL; Pfizer Pharmaceuticals, Sandwich, UK) and ketamine (100 mg/mL; Pfizer Pharmaceuticals) mixed with sterile water in a 5:3:42 ratio. If the procedure was not terminal, anesthesia was reversed using i.p. administration of 0.2 mL of Antisedan (atipamezole; Pfizer Pharmaceuticals).

### Induction of CNV and Fluorescein Angiography

After the induction of anesthesia, CNV was induced using rupture of the Bruch's membrane using a slit-lamp–mounted diode (wavelength 680 nm) laser system (Keeler, Windsor, UK) with the following settings: 210 mW power; 100 ms duration; 100 μm spot diameter. These settings consistently generated a subretinal gas bubble indicative of rupture of the Bruch's membrane and successful induction of CNV.^25^ Three laser burns were delivered (two to three disc diameters away from the optic nerve head at the 2-, 6-, and 10-o'clock positions, avoiding any vessel) to both eyes of 6- to 12-week-old mice with the slit-lamp–mounted diode laser system (wavelength 680 nm; Keeler). Eyes with presenting signs of hemorrhage, cataract, or keratopathy formation were excluded. Lesions were excluded if laser-induced subretinal bubble formation did not occur. Animals were excluded if choroidal hemorrhage after laser-induced injury (as this occurrence may have affected the development or measurement of neovascularization) or significant cataract or keratopathy (as these lesions may have obscured fundus view, hindering laser energy delivery or angiography) was present.

At days 7 and 14 after laser-induced injury, images of choroidal lesions were acquired on tropicamide-dilated eyes using a 55-degree–angle lens on high-resolution and automated real-time modalities. *In vivo* fluorescein angiography was performed using acquisition of early-phase images at 90 seconds after i.p. administration of 0.2 mL of fluorescein solution (20 mg/mL) via the HRA2 confocal scanning laser ophthalmoscope (Heidelberg Engineering, Hemel Hempstead, UK) in fundus autofluorescence mode (excitation 488 nm, emission 500 to 700 nm). CNV lesions were quantified using ImageJ software version 1.54c (NIH, Bethesda, MD; *https://imagej.nih.gov/ij*). The analyzer (E.C.) was masked to the genotype of the samples.

### *In Vivo* Autofluorescence Imaging

Autofluorescence imaging was performed using the HRA2 confocal scanning laser ophthalmoscope as described in Induction of CNV and Fluorescein Angiography. The near-infrared reflectance mode (820 nm laser) was used for alignment and to focus at the subretinal space, after which the confocal scanning laser ophthalmoscope was switched to fundus autofluorescence mode.

### Preparation of Whole-Mounts and Immunofluorescence

After animal sacrifice, eyes were fixed in 4% (w/v) paraformaldehyde for 2 hours and choroid/RPE whole-mounts were prepared as described.^10^^,^^25^ After blocking in 5% (v/v) normal goat serum, 1% (w/v) bovine serum albumin, 1% (v/v) Triton X-100 phosphate-buffered saline 1X, resident immune cells were stained using incubation with rabbit anti–ionized calcium-binding adaptor molecule 1 primary antibody (1:1000 in block buffer; Alpha Laboratories, Eastleigh, UK) overnight at 4°C, followed by Alexa Fluor 488–conjugated anti-rabbit IgG secondary antibody (1:500 in block buffer; Thermo Fisher Scientific, Horsham, UK) incubation for 2 hours at room temperature; each step was followed by extensive washing in 1% (v/v) Triton X-100 phosphate-buffered saline 1X. For cytoskeleton staining with Alexa Fluor 546–conjugated Phalloidin (1:40 in block buffer; Thermo Fisher Scientific), incubation was performed for 30 minutes at room temperature, followed by extensive washing. Vasculature and CNV lesions were visualized using overnight incubation at 4°C with biotin-conjugated *Bandeiraea simplicifolia* isolectin B4 (Sigma-Aldrich, Gillingham, UK), followed by Alexa Fluor 633–conjugated streptavidin incubation for 2 hours at room temperature.

### Visualization of the Choroidal Microvasculature

Choroidal vasculature was visualized as described previously.^26^ Briefly, mice were transcardially perfused with 10 mL of oxygenated Ringer solution containing heparin, followed by 4 mL of fluorescein isothiocyanate–conjugated *Griffonia simplicifolia* isolectin B4 (0.1 mg/mL; 2BScientific, Kirtlington, UK) and 10 mL of phosphate-buffered saline 1X, to remove unbound ligand. RPE choroidal tissue was then flat-mounted and incubated for 2 minutes in 0.25% trypsin (Thermo Fisher Scientific). Gentle brushing and pipetting were applied to remove pigmented RPE cells and facilitate the visualization of choroidal vasculature.

### Imaging and Quantification of Cellular Features

Choroid/RPE flat mounts were imaged on a confocal microscope (Leica TCS SPE, Leica Microsystems, Milton Keynes, UK). Mosaic stack images were generated to visualize the entire tissue or lesion at 512 × 512-pixel resolution with a 10× dry objective. Stacks were then Z-projected on proprietary software (Leica LAS AF version 4.0). Data from at least four regions of interest/eye were quantified and averaged, for both the central and peripheral portions of each sample, excluding scarred depigmented areas, if present, which were mostly devoid of cells. Quantifications of cellular morphology of RPE monolayer (cell number per square millimeter; cell area and perimeter, number of myeloid cells per square millimeter of subretinal space) and choroidal microvasculature features were performed on ImageJ. To measure CNV three-dimensional lesions, fine confocal *z*-stacks of isolectin B4–stained choroidal tissue were three-dimensionally reconstructed, surface rendered, and volume measured using Imaris software version 8 (Bitplane, Schlieren, Switzerland). The analyzer (E.C.) was masked to the genotype of the samples.

### Real-Time PCR

Choroid/RPE tissue was manually dissected and homogenized by using RNA extraction buffer obtained from the RNeasy Mini Kit (Qiagen, Manchester, UK), which was subsequently used to extract total RNA. cDNA was prepared using the QuantiTect Reverse Transcription Kit (Qiagen). Relative quantification of genes of interest was performed using real-time probe-based PCR against endogenous β-actin expression levels as described.^10^ Real-time PCR master mix was used by following the recommended guidelines (Quantabio, Beverly, MA).

### *In Vitro* Choroid Sprouting Assay

*Ex vivo* choroid sprouting assay was performed as described with modifications.^27^ Dissected (postnatal days 18 to 21) peripheral choroid samples were cut into approximately 1 × 1-mm pieces and placed in growth factor–reduced Matrigel (BD Biosciences, Plymouth, UK) in 24 well plates. After seeding, Matrigel was left to polymerize at 37°C for 10 minutes in 500 μL of the complete microvascular endothelial cell growth medium EGM-2MV BulletKit (Lonza, Little Chesterford, UK) added in each well, and explants were incubated at 37°C, 5% CO_2_ to acclimatize overnight. Next day, 0.5% O_2_ hypoxia regimen was initiated in a dedicated cell culture incubator (Wolflabs, Pocklington, UK) by N_2_ exchange, with cell culture medium exchanged every third day. Sprouting area was imaged at days 3, 4, 5, and 6 and quantified using the SWIFT-Choroid computerized ImageJ plug-in, with permission granted from Shao et al.^27^

### Statistical Analysis

Data were plotted and analyzed using Prism software version 5 (GraphPad Software Inc., San Diego, CA) and are presented as means ± SEM. Normal distribution in each group was assessed using the Kolmogorov-Smirnov test. A suitable parametric statistical test (one- or two-way analysis of variance) was used to compare data between more than two groups, with the *post hoc* Tukey or Bonferroni test for multiple comparisons. *P* < 0.05 was considered statistically significant.

## Results

### Characterization of Cre-Mediated Recombination Efficiency at the RPE/Choroid Level

**Figure 1:**
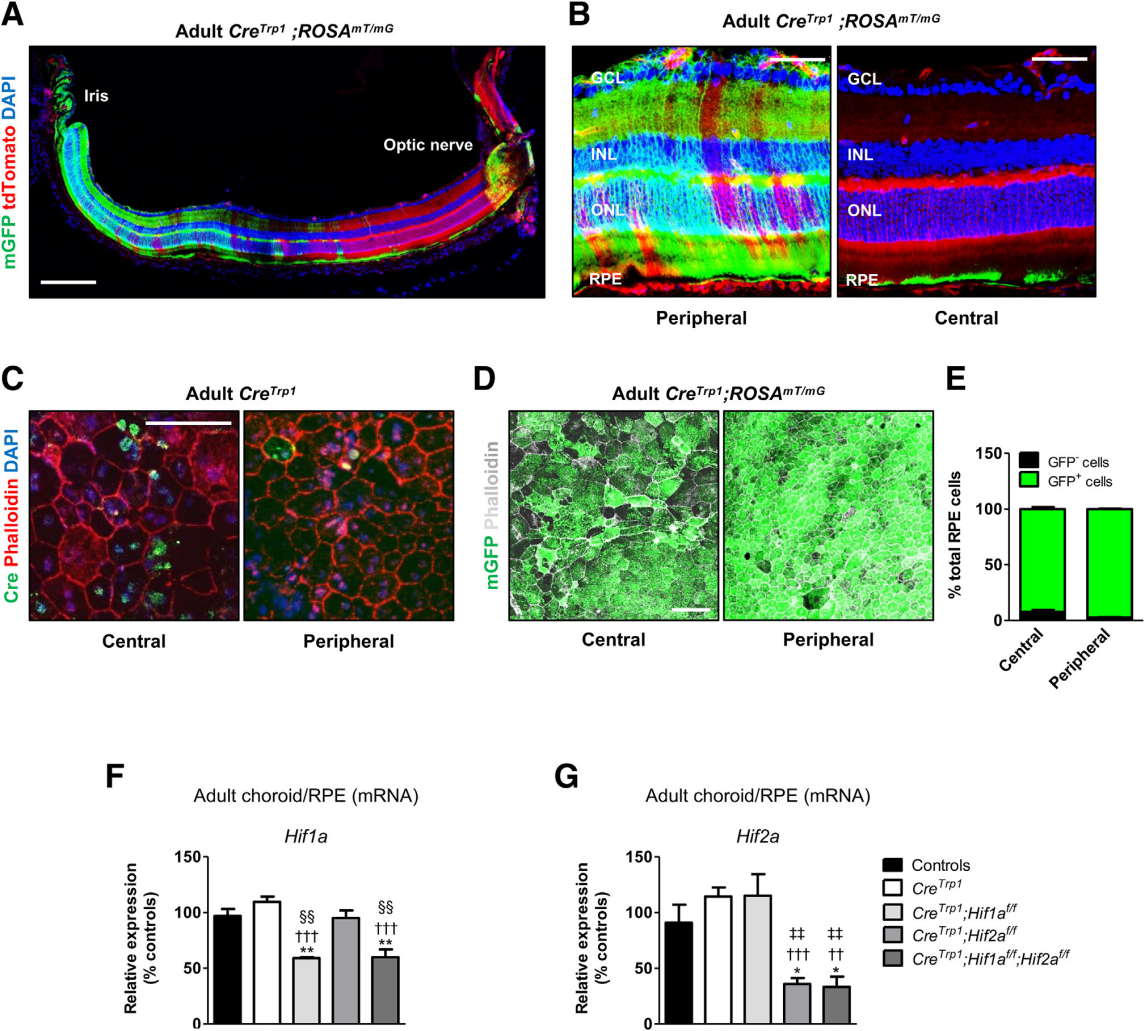
Characterization of Cre-mediated recombination and HIF expression in RPE cells of the *Cre*^*Trp1*^*;Hif*_x_^*f/f*^ lines. **A:** Representative eye section from an adult *Cre*^*Trp1*^*;ROSA*^*mT/mG*^ mouse, showing membrane-targeted Green fluorescent protein (mGFP) distribution in the different cellular layers. Cre-mediated excision in the neuroretina of *Cre*^*Trp1*^ mice has been reported previously.^10^**B:** Central and peripheral confocal magnified images show mGFP expression (and Cre-mediated DNA excision) to be confined to the RPE monolayer in the RPE/choroid/sclera ocular compartment. **C:** Representative confocal images of central and peripheral RPE/choroid flat mount from an adult *Cre*^*Trp1*^ mouse indicate persistent Cre expression in adulthood. **D:** Representative images of central and peripheral portions of RPE/choroid flat mounts from adult *Cre*^*Trp1*^*;ROSA*^*mT/mG*^. tdTomato fluorescence not shown. **E:** Quantification of central and peripheral RPE cells stained positive for mGFP (ie, derived from cells that underwent Cre-mediated recombination). **F** and **G:** RT-qPCR analysis of whole adult RPE/choroid samples from the reported genotypes; targets analyzed are *Hif1a* (**F**) and *Hif2a* (**G**). Data are expressed as means ± SEM. *n* = 4 to 5 animals per genotype (**F** and **G**); *n* = 5 to 6 animals per genotype (**E**). ∗*P* < 0.05, ∗∗*P* < 0.01 versus controls in the same experiment (analysis of variance and Tukey test for multiple comparisons); ^††^*P* < 0.01, ^†††^*P* < 0.001 versus *Cre*^*Trp1*^; ^‡‡^*P* < 0.01 versus *Cre*^*Trp1*^*;Hif1a*^*f/f*^; ^§§^*P* < 0.01 versus *Cre*^*Trp1*^*;Hif2a*^*f/f*^ (all, versus group in the same experiment). Scale bars: 200 μm (**A**); 50 μm (**B** and **C**); 100 μm (**D**). GCL, ganglion cell layer; INL, inner nuclear layer; ONL, outer nuclear layer.

Despite gene homozygosity, mosaicism of Cre expression in the RPE of the *Cre*^*Trp1*^ line has been described previously.^11^ To further characterize the expression specificity and efficiency of Cre-mediated recombination, *Cre*^*Trp1*^ was crossed with the reporter *ROSA*^*mT/mG*^ line, whose ubiquitous membrane-bound tdTomato expression switches to membrane-targeted Green fluorescent protein (mGFP) expression by Cre-mediated excision.^23^ Ectopic *Cre* expression in the neuroretina, due to *Tyrp1* promoter activation in the retina progenitor cells, has been reported in *Cre*^*Trp1*^.^10^ These findings were confirmed using the detection of neuroretinal mGFP expression in 4- to 6-week–old *Cre*^*Trp1*^*;ROSA*^*mT/mG*^ mice almost exclusively in the peripheral retina (Figure 1, A and B). In the choroid/RPE/sclera ocular compartment, Cre-mediated excision was detected solely at the RPE level (Figure 1A), both centrally and peripherally (Figure 1C). Evidence of RPE Cre protein expression in young adult *Cre*^*Trp1*^ mice (Figure 1C) indicated that *Tyrp1* promoter activity is sustained for longer than previously reported (postnatal day 12).^9^^,^^11^

In adult choroid/RPE flat mounts, mGFP expression was detected consistently in almost 100% of RPE cells, both centrally and peripherally (Figure 1, D and E), indicating efficient *Cre* expression and Cre-mediated recombination of the *tdTomato/GFP* reporter system in the large majority of the pigmented epithelial cells, in contrast to the mosaic expression reported previously by utilizing a *LacZ* reporter system.^11^ However, given that Cre*-*mediated recombination is influenced by the chromosomal location of the *loxP*-flanked (floxed) gene,^28^ the excision efficiency of two different floxed loci was evaluated in adult choroid/RPE samples by crossing the *Cre*^*Trp1*^ with the *Hif1a*^*f/f*^ and *Hif2a*^*f/f*^ lines. *Hif1a* expression was significantly reduced in *Cre*^*Trp1*^*;Hif1a*^*f/f*^ and *Cre*^*Trp1*^*;Hif1a*^*f/f*^*;Hif2a*^*f/f*^ mice (40% reduction compared to *Cre*-negative controls) (Figure 1F), whereas *Hif2a* expression was reduced in *Cre*^*Trp1*^*;Hif2a*^*f/f*^ and *Cre*^*Trp1*^*;Hif1a*^*f/f*^*;Hif2a*^*f/f*^ mice (65% and 67% reductions, respectively, compared to *Cre*-negative controls) (Figure 1G). These findings confirmed how *Cre*^*Trp1*^-mediated recombination efficiency depends on the location of the *loxP*-flanked transgene and suggests the possibility of a differential chromatin density–dependent access by Cre recombinase in RPE cells located in different regions.

### *Cre*^*Trp1*^ Abnormalities in the RPE Monolayer Are Reduced in the Absence of HIF1α and Exacerbated in the Absence of HIF2α

**Figure 2:**
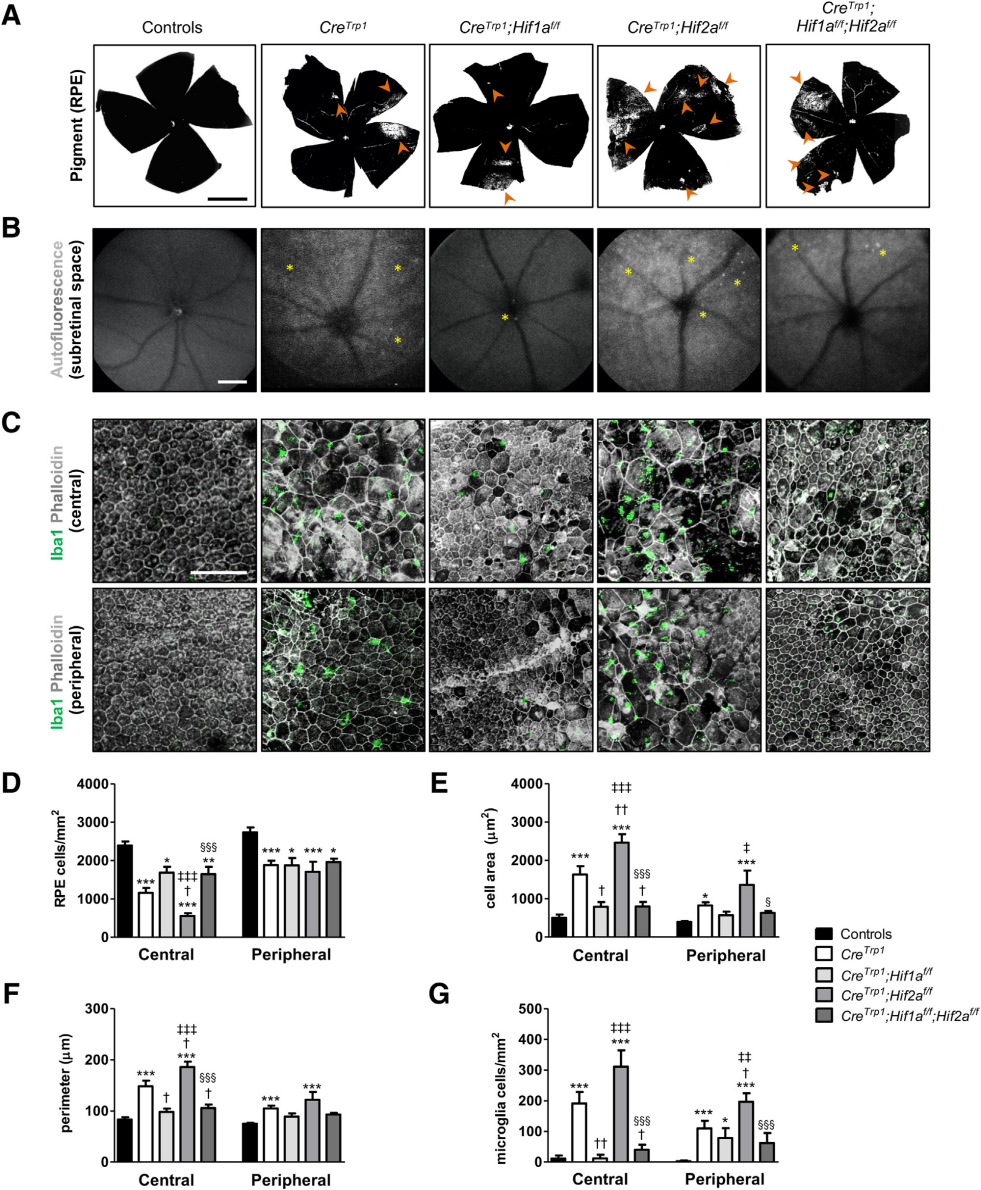
Morphologic abnormalities of RPE monolayer in *Cre*^*Trp1*^*;Hif*x^*f/f*^ lines. **A:** Bright-field images of adult choroid/RPE flat mounts; examples of depigmented areas are highlighted by **arrowheads**. **B:** Representative live autofluorescence scanning laser ophthalmoscopy images in adult animals, focusing on the subretinal space/RPE region and highlighting qualitative differences in diffused hyper-reflective regions and hyper-reflective foci (**asterisks**) among the *Cre*^*Trp1*^ lines. **C:** Immunohistochemistry staining of phalloidin and ionized calcium-binding adaptor molecule 1 (Iba1) (both central and peripheral regions) show a particularly severe morphologic phenotype in adult RPE cells and ameboid-shaped inflammatory cells migrated in the subretinal space in correspondence to damaged RPE regions in *Cre*^*Trp1*^ and *Cre*^*Trp1*^*;Hif2a*^*f/f*^ mice. **D–G:** Quantification of cell density (**D**), cell area (**E**), cell perimeter (**F**), and Iba1^+^ cell density (**G**) in both the peripheral and central RPE portions. Data are expressed as means ± SEM. *n* = 5 to 30 animals per genotype. ∗*P* < 0.05, ∗∗*P* < 0.01, and ∗∗∗*P* < 0.001 versus controls; ^†^*P* < 0.05, ^††^*P* < 0.01 versus *Cre*^*Trp1*^; ^‡^*P* < 0.05, ^‡‡^*P* < 0.01, and ^‡‡‡^*P* < 0.001 versus *Cre*^*Trp1*^*;Hif1a*^*f/f*^; ^§^*P* < 0.05, ^§§§^*P* < 0.001 versus *Cre*^*Trp1*^*;Hif2a*^*f/f*^ (all, versus groups in the same RPE region, by analysis of variance and Tukey test for multiple comparisons). Scale bars: 1 mm (**A** and **B**); 100 μm (**C**).

One of the characteristic features of the *Cre*^*Trp1*^ line is the pigmentary defects visible macroscopically in RPE/choroid flat mounts.^11^ All of the *Cre*^*Trp1*^-based lines employed in this study showed areas of depigmentation primarily in the periphery of the tissue, suggestive of cellular dysfunction and atrophy (Figure 2A and Supplemental Figure S1). *Cre*^*Trp1*^*;Hif2a*^*f/f*^ showed a qualitatively more severe phenotype with more prominent and extensive areas of depigmentation affecting primarily the periphery but also the central portion of the RPE monolayer, whereas *Cre*^*Trp1*^*;Hif1a*^*f/f*^ showed a less severe phenotype (*Cre*^*Trp1*^*;Hif1a*^*f/f*^ and *Cre*^*Trp1*^*;Hif2a*^*f/f*^) (Figure 2A).

The autofluorescence signal by confocal scanning laser ophthalmoscopy was utilized to detect fundus changes. Adult *Cre*^*Trp1*^ mice showed diffuse hyper-fluorescence and autofluorescent spots in the outer retina/subretinal space (Figure 2B), which have been shown to signify tissue dysfunction, degeneration, and inflammatory cascade activation.^26^ Notably, the *Cre*^*Trp1*^*;Hif1a*^*f/f*^ phenotype was similar to that observed in control lines, with reduced autofluorescence and fewer spots visible than in *Cre*^*Trp1*^ and other *Cre*^*Trp1*^-based lines. In contrast, the *Cre*^*Trp1*^;*Hif2a*^*f/f*^ phenotype was more severe than that of *Cre*^*Trp1*^, with more intense hyper-fluorescence and qualitatively more autofluorescent spots. The double-knockout *Cre*^*Trp1*^*;Hif1a*^*f/f*^*;Hif2a*^*f/f*^ line showed a phenotype less severe than *Cre*^*Trp1*^*;Hif2a*^*f/f*^.

Severe alterations in RPE monolayer morphology and structure of the *Cre*^*Trp1*^ line have previously been described.^11^ *Cre* expression in the RPE leads to loss of the typical honeycomb cellular structure, a reduction in cell density, and an associated increase in cell size and perimeter, particularly in the central portion of the tissue; in addition, ameboid-shaped phagocytic myeloid cells are evident in areas of dysfunctional/altered RPE. Beyond confirmation of these observations (Figure 2, C–G), *Hif1a* ablation was observed to be ameliorated all of the quantified parameters, producing a cellular phenotype more closely resembling that of controls. In contrast, when *Hif2a* expression was reduced, the cellular phenotype appeared more severe than that observed in the *Cre*^*Trp1*^ line, mainly at the center. In the double-knockout, *Hif1a* ablation attenuated the severity of the phenotype seen in the *Hif2a* knockout line (KO) to values closer to those seen in control lines.

### Choroidal Vasculature Organization Is Affected by the RPE Monolayer Defects

**Figure 3:**
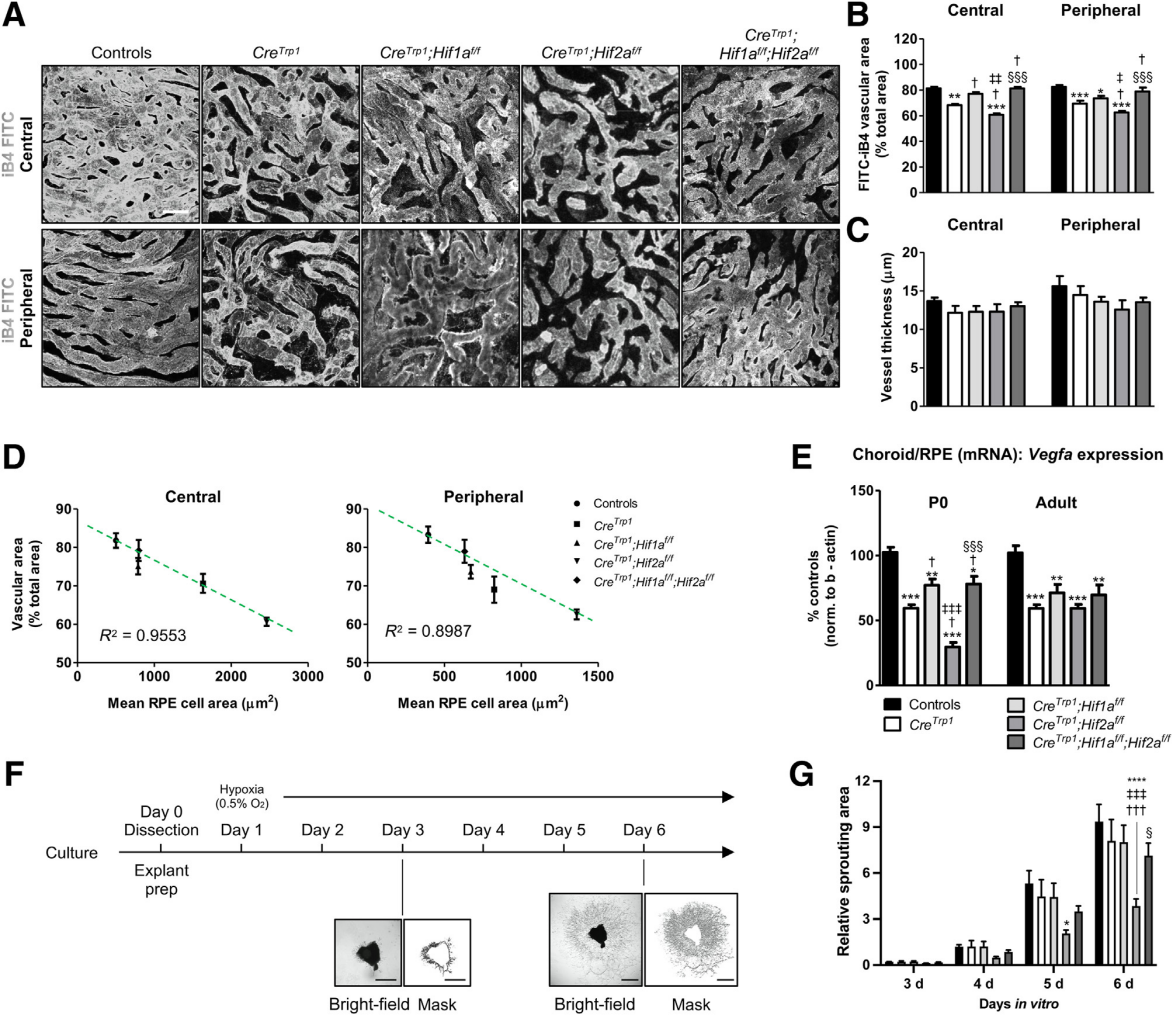
Choroidal vascular phenotype in *Cre*^*Trp1*^*;Hif*_x_^*f/f*^ lines. **A:** Confocal images of isolectin B4–fluorescein isothiocyanate (iB4-FITC)-perfused adult choriocapillaris in the phenotypes under analysis. Representative examples in central and peripheral portions are reported. **B** and **C:** Quantification of vascularized area (**B**) and vessel thickness (**C**). **D:** Between-genotype comparisons of mean RPE cell area and choroidal vascularized area, showing negative linear correlation. Linear interpolation equations: central, *y* = –0.0101*x* + 86.001; peripheral, *y* = –0.0214*x* + 90.148. **Dashed lines** represent the calculated data interpolation lines. Coefficients of determination (*R*^2^) are shown (the closer to 1.0, the more reliable the fit of the model). **E:** RT-qPCR analysis of *Vegfa* expression in whole P0–P1 and adult RPE/choroid samples. Genotypes in **E** are applicable to **B**, **C**, and **G**. **F:***In vitro* choroidal sprouting assay experiment and representative examples of choroidal endothelium growth at 3 and 6 days *in vitro*. P18-P21 eyes were dissected in 1 × 1-mm explants, embedded in Matrigel, and placed in culture for 6 days under deep hypoxic conditions. Sprouting was assessed microscopically on a daily basis from day 3. **G:** Quantification of sprouting area over time. For simplicity, data from days 0 to 2 (no detectable sprouting) are not shown. Data are expressed as means ± SEM. *n* = 3 to 5 explants per animal (**G**); *n* = 4 to 7 animals per genotype (**G**); *n* = 4 to 14 animals per genotype (**E**); *n* = 5 to 18 animals per genotype (**D**). ∗*P* < 0.05, ∗∗*P* < 0.01, ∗∗∗*P* < 0.001, and ∗∗∗∗*P* < 0.0001 versus controls; ^†^*P* < 0.05, ^†††^*P* < 0.001 versus *Cre*^*Trp1*^; ^‡^*P* < 0.05, ^‡‡^*P* < 0.01, and ^‡‡‡^*P* < 0.001 versus *Cre*^*Trp1*^*;Hif1a*^*f/f*^; ^§^*P* < 0.05, ^§§§^*P* < 0.001 versus *Cre*^*Trp1*^*;Hif2a*^*f/f*^ (**C,** versus groups in the same choroidal region, by analysis of variance and Tukey test for multiple comparisons; **E,** versus groups of the same age, by analysis of variance and Tukey test for multiple comparisons; **G,** versus groups cultured for the same number of days *in vitro*, by analysis of variance and Bonferroni test for multiple comparisons)*.* Scale bars: 25 μm (**A**); 1 mm (**F**).

One of the roles played by the RPE monolayer is to support the development, maintenance, and function of the choroidal vasculature.^3^^,^^4^^,^^29^ Areas of RPE atrophy/absence are associated with choriocapillaris dropout in both mice^3^ and humans.^7^ The choroidal vasculature was examined using transcardial perfusion of fluorescein isothiocyanate–conjugated isolectin and detected reduced vascular density in adult *Cre*^*Trp1*^ mice, both centrally and peripherally (Figure 3, A and B). Interestingly, such phenotype was less pronounced in *Hif1a*-ablated mice (the difference versus controls in the central retina portion was non-significant), and was more exacerbated in *Hif2a*-KO lines, both centrally and peripherally. However, the concomitant ablation of *Hif1a* ameliorated such phenotype. No differences were detected in terms of vessel thickness (Figure 3C). Notably, the mean choroidal vascular density of all investigated genotypes correlated negatively, with a high degree of confidence, with the mean RPE cell area (*R*^2^ ≥ 0.898), which was used as an indicator of abnormal cellular morphology in the RPE (Figure 3D).

The angiomodulatory protein vascular endothelial growth factor (VEGF)-A has been described as one of the major molecular signals produced by the RPE to support physiological choroidal vasculature development.^3^^,^^4^^,^^29^ Both HIF1α and HIF2α are potent *Vegfa* transcriptional regulators during development and pathology; however, they often drive nonoverlapping and unique responses to hypoxia.^10^^,^^16^ To determine whether differences in *Vegfa* expression could account for HIF-related vascular defects, *Vegfa* expression was measured in neonatal (P0) and adult choroid/RPE using real-time quantitative (q)-PCR (Figure 3E). Reduced *Vegfa* expression in all *Cre*^*Trp1*^ lines was detected in both age groups. In the neonatal group specifically, *Vegfa* expression was more strongly reduced in the *Hif2a*–single-KO line than in the *Hif1a*-KO line. Given that the neonatal group was likely to represent more closely the low oxygen conditions *in utero* during late prenatal ocular development, these findings are consistent with the involvement of VEGF-A during choroidal vasculature development,^4^ indicating HIF2α as a key transcriptional inducer and HIF1α as a negative dominant regulator of *Vegfa* expression in the RPE.

To confirm the link between abnormal RPE morphology, reduced choroidal vascular formation, and involvement of HIFs, choroid/RPE explants were cultured from different genotypes *ex vivo* under deep hypoxic conditions (0.5% O_2_) and were monitored for choroidal endothelial cell sprouting (Figure 3F). *Cre*^*Trp1*^ and *Cre*^*Trp1*^;*Hif1a*^*f/f*^ explants grew comparably to the controls, whereas *Cre*^*Trp1*^;*Hif2a*^*f/f*^ showed a reduced vascularization, which could be restored to control levels by concomitant *Hif1a* ablation (Figure 3G). These findings resembled those observed in immune-stained flat mounts (Figure 3, A and B).

### HIF*-*Dependent Responses to Laser CNV in the *Cre*^*Trp1*^ Mouse

**Figure 4:**
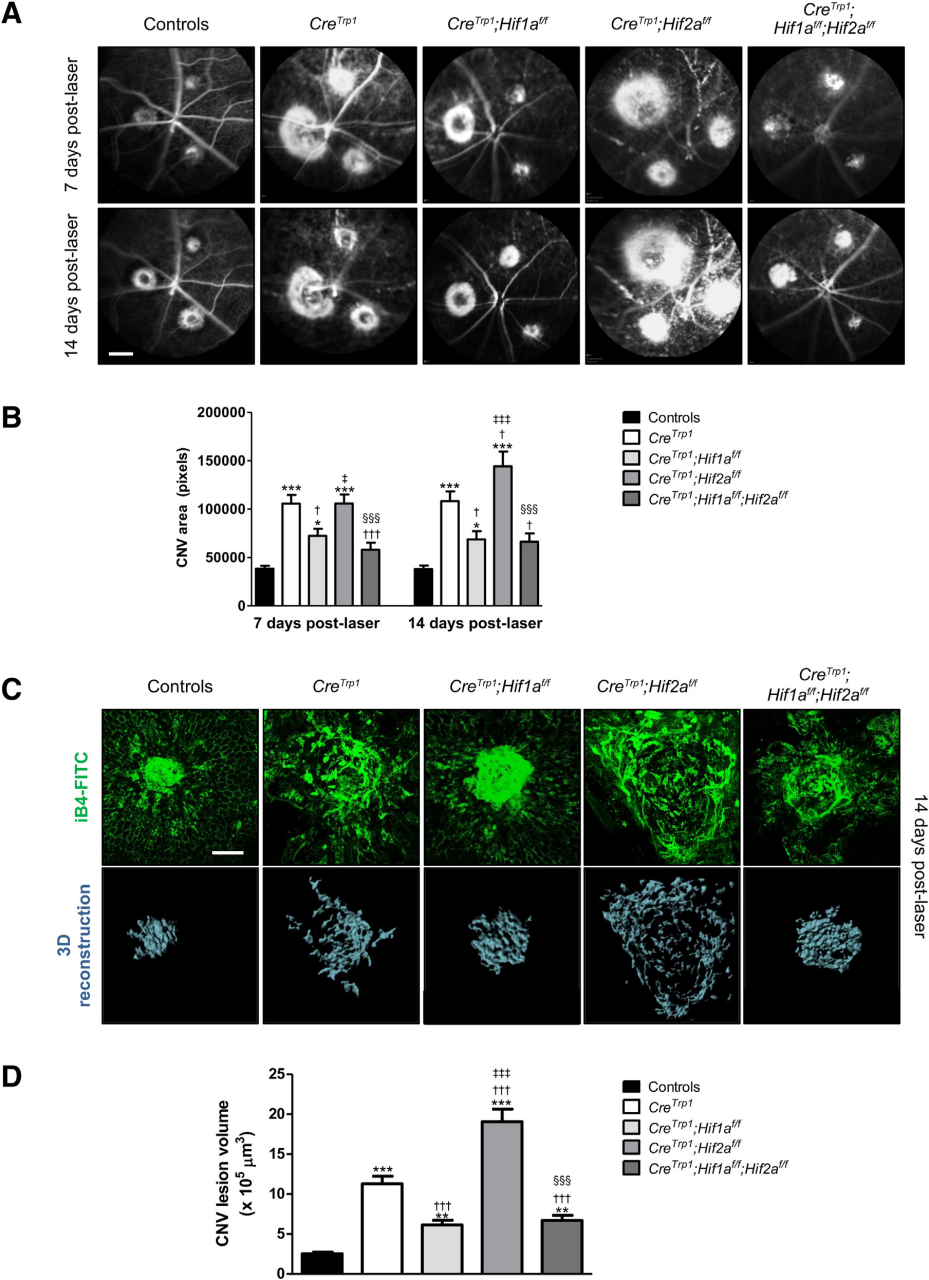
Differences in laser choroidal neovascularization (CNV) lesions in *Cre*^*Trp1*^*;Hif*x^*f/f*^ lines at days 7 and 14 after laser-induced injury. **A:** Representative images of laser vascular injuries visualized using fundus fluorescein angiography. **B:** Quantification of CNV lesion area. **C:** Representative confocal images of laser vascular injuries stained with isolectin B4 at day 14 after laser injury and three-dimensional (3D) reconstruction. **D:** Quantification of CNV lesion volumes at day 14 using Imaris software version 8. Data are expressed as means ± SEM. *n* = 14 to 45 laser burns per genotype. ∗*P* < 0.05, ∗∗*P* < 0.01, and ∗∗∗*P* < 0.001 versus controls; ^†^*P* < 0.05, ^†††^*P* < 0.001 versus *Cre*^*Trp1*^; ^‡^*P* < 0.05, ^‡‡‡^*P* < 0.001 versus *Cre*^*Trp1*^*;Hif1a*^*f/f*^; ^§§§^*P* < 0.001 versus *Cre*^*Trp1*^*;Hif2a*^*f/f*^ (all, versus groups analyzed at the same number of days after laser injury, by analysis of variance and Tukey test for multiple comparisons). Scale bars: 2 mm (**A**); 100 μm (**C**). iB4-FITC isolectin B4–fluorescein isothiocyanate.

RPE dysfunction, degeneration, and malfunction can predispose to CNV in AMD.^30^ To investigate the roles of HIF1 and HIF2 in this process, the effects of *Hif1a* and *Hif2a* ablation were measured on laser-induced CNV in *Cre*^*Trp1*^ mice. At day 7 after laser-induced injury, *in vivo* fluorescein angiography identified significantly larger lesions in *Cre*^*Trp1*^ and *Cre*^*Trp1*^*;Hif2a*^*f/f*^ eyes compared to all other genotypes, whereas at day 14 after laser-induced injury, *Cre*^*Trp1*^*;Hif2a*^*f/f*^ lesions were found to be significantly larger than those developed by *Cre*^*Trp1*^. Interestingly, the ablation of *Hif1a* led to significantly smaller lesions than those measured in both the *Cre*^*Trp1*^ and the *Cre*^*Trp1*^*;Hif2a*^*f/f*^ lines at both time points (Figure 4, A and B).

Findings on fluorescein angiography may be affected by *in vivo* dye extravasation; to measure with greater-precision differences in lesion size, CNV lesions at day 14 after laser-induced injury were stained with isolectin B4, which labels both vascular endothelial cells and myeloid cells such as microglia. Tissue was imaged using confocal microscopy and digitally reconstructed in three dimensions to measure the volume of the lesion formed of neovessels and recruited microglia. The same differences identified using fluorescein angiography *in vivo* at day 14 after laser-induced injury were statistically confirmed (Figure 4, C and D).

## Discussion

RPE-specific Cre recombinase lines have inherent limitations.^11^ Besides showing patchy recombination, prolonged postnatal Cre expression, and lack of cell specificity,^10^^,^^20^ the widely utilized *Cre*^*Trp1*^ line is characterized by dose-dependent Cre-mediated toxicity, leading to dramatic morphologic changes in the RPE monolayer, pigmentation defects, choroidal vessel dropout, and activation of the innate immunity, which are all features similar to the changes that occur during the early stages of AMD. In the present study, the role of the HIF pathway, which is highly active during embryogenesis and prenatal development,^31^^,^^32^ was investigated in this Cre-mediated degeneration for insights that may be relevant to macular degeneration.

Despite sustained (Figure 1C) and widespread (Figure 1, A, B, D, and E) expression and activity of Cre^9^^,^^11^ when evaluated using the means of the *tdTomato/GFP* reporter system, the *Cre*^*Trp1*^ line showed transgene locus- and RPE cellular location–dependent variability in recombination efficiency and gene inactivation potentially due to differences in chromatin accessibility (Figure 1, D–G), a limitation previously described in the *Cre/loxP* system.^28^ The *Cre*^*Trp1*^ line showed changes in the RPE pigmentation and increased subretinal autofluorescence, which are indicators of lipofuscin accumulation and RPE oxidative damage in humans,^33^ presence of hyper-autofluorescent spots, which is suggestive of immune cell infiltrates (as confirmed using immunostaining) and/or nonrecycled photoreceptor outer segments,^34^ and alterations in the numbers and typical RPE cellular morphology. This phenotype was found to impact differentially the central and peripheral regions of the RPE, which are known to present intrinsic differences in terms of survival, proliferation, and morphologic adaptation.^29^^,^^35^ Although depigmentation was qualitatively observed in both portions of the RPE monolayer (Supplemental Figure S1), the peripheral portion showed more signs of macroscopic depigmentation/end-stage tissue scarring (Figure 2A) than did the central portion, which demonstrated a more severe phenotype at a cellular morphology level (Figure 2, C–G) and resembled the RPE atrophy evident in human eyes affected by atrophic AMD.^36^ These morphologic changes are consistent with compensatory responses by surviving cells to maintain a tight monolayer.

Both peripherally and centrally, the choroidal vasculature was found to be less dense than in control lines (Figure 3), and there was a negative correlation between RPE cell area and vascularized choroid, consistent with the trophic role of the RPE on the underlying vasculature.^4^^,^^29^

The conditional ablation of *Hif1a* partially improved the phenotype from a morphologic and vascular perspective (Figures 2 and 3). In contrast, the ablation of *Hif2a* led to more severe features, which could be mitigated by the concomitant knockout of *Hif1a*. These findings indicate that HIF1α is a mediator of the atrophic changes in the *Cre*^*Trp1*^ line, whereas HIF2α plays a protective role. This evidence offers further insight on the role of HIFs in mediating RPE structural changes^37^ and on the distinct, nonredundant responses to low oxygen levels by the two main HIFs.^16^^,^^35^^,^^38^ The role of the third member of the family, HIF3α, in mediating and regulating the RPE toxic response to Cre was not investigated here but warrants further attention given that conditional *Hif1a* and *Hif2a* ablation could explain only part of the phenotype.

Cre recombinase is known to cause cellular toxicity in a time- and dose-dependent manner^39^ by nonspecific genomic recombination at loci of limited homology with the 34-bp *loxP* sequence. Despite being postnatally a postmitotic quiescent tissue with less accessible chromatin, the RPE appears to be particularly susceptible to genotoxicity and morphologic changes after Cre recombinase activation, even after time-limited postnatal Cre expression.^39^ Notably, despite the random nature of these genomic occurrences, the morphologic outcomes at the outer retina barrier level appear to be strikingly consistent and reproducible among lines used in this and other studies. This finding points toward the activation of a specific molecular response to the aberrant genomic events, likely resulting in impaired tissue development, cell dysfunction/de-differentiation, and eventually cell death.^13^^,^^40^ Interestingly, HIF1α (but not HIF2α) was found to be linked to genetic instability and inhibition of DNA repair in cancer models.^41^ Other relevant pathways regulated differently or exclusively by one of these transcription factors are tight-junction integrity and epithelial cellular morphology,^42^^,^^43^ as well as energy metabolism and apoptosis,^19^ which support the hypothesis of HIFs being involved in mediating the degenerative process seen in the *Cre*^*Trp1*^ line.

To explain the abundant presence of activated microglia in the subretinal space of the *Cre*^*Trp1*^ line, Thanos et al^11^ found decreased levels of the immunosuppressive cytokine IL-10 in RPE/choroid protein extracts and hypothesized a reduced expression of other immunosuppressive mediators such as transforming growth factor β and pigment epithelium–derived factor. Local hypoxia and inflammation, by means of HIF1α and HIF2α, are known to modulate the expression of these cytokines,^44^, ^45^, ^46^, ^47^ providing an interesting insight on how the specific deletion of either factor may lead to significantly different outcomes from a local immunity perspective. This hypothesis may also account for the results presented here for both RPE/vascular development (Figure 2, C–G) and CNV (Figure 4).

During both development and aging, the RPE and the choroidal vasculature are tightly interlinked, each contributing to the formation, maintenance, and malfunction of the other.^3^^,^^4^^,^^7^^,^^37^ With regard to developmental choroidal vascularization, a correlation between the severity of the vascular phenotypes in the different lines and the expression of VEGF-A in neonatal choroid/RPE samples was observed (Figure 3E). Under deep prenatal hypoxic conditions, this potent angiogenic effector is modulated by HIF1α and HIF2α differently, as previously shown in the RPE and other ocular tissues.^4^^,^^10^ In this specific context, the results presented in this article point toward HIF1α being a negative modulator, and HIF2α, a positive modulator, of VEGF-A expression, in contrast to previous suggestions.^4^ Interestingly, the adult choroidal/RPE expression of VEGF-A, known to be a fundamental trophic factor to the choroidal vasculature in adult mice,^29^ remained significantly lower but was comparable in all *Cre*^*Trp1*^-based lines, which points toward prenatal hypoxia-related mechanisms and not to a previously suggested^4^ lack of post-developmental vascular trophic support to explain the observed phenotype. In addition, alongside a marked reduction in VEGF-A expression, the authors have previously reported reduced erythropoietin and increased endostatin (a potent antiangiogenic factor) in *Cre*^*Trp1*^*;Hif2a*^*f/f*^ retinae and retinal neuroprogenitors involved in guiding retinal vasculature formation postnatally in the murine model.^10^ Given the similarities between neuroprogenitors and RPE cells in providing trophic and developmental support to the formation of the vascular beds of the respective tissues, it is speculated that HIFs may differentially drive similar molecular pathways.

The laser-induced choroidal murine model has been widely exploited to produce important pathophysiological and translational findings in CNV, although it is artificial in nature and lacks some of the features seen in neovascular AMD.^48^ Interestingly, the *Cre*^*Trp1*^ does present some key features that render it closer to the early stages of the human pathology, namely choroidal vessel dropout, dysfunctional/atrophic RPE, and significant activation of the local innate immunity, all known to contribute to choroidal vascular lesion formation,^30^ which led the authors to utilize this experimental model in the genotypes under investigation. Increased expression and stabilization of HIFs, resulting from age-related RPE changes and hypoxia, have been detected in tissue from patients with neovascular age-related macular degeneration,^49^ and their inhibition (in particular of HIF1α) has previously been pursued as a novel therapeutic strategy.^29^^,^^50^ Interestingly, in the present model, *Hif1a* deletion protected against lesion formation in the already degenerating *Cre*^*Trp1*^ eye, whereas *Hif2a* inhibition exacerbated the phenotype. These findings differ in part from those of a previous report in a postnatal Cre-inducible RPE-specific murine model,^29^ which lacks the degenerative features seen instead in the *Cre*^*Trp1*^ line. Overall, these results highlight clear differences between physiological (mainly hypoxia-driven) and pathologic (mainly inflammation-driven) choroidal angiogenesis, which in both cases see HIFs playing significant roles.

In conclusion, by further describing the morphologic features of the *Cre*^*Trp1*^ line and the involvement of HIF1α and HIF2α, this model provides insight into the physiopathology of RPE degeneration and CNV under innate immunity and hypoxia signaling and may serve for the testing of relevant therapeutic approaches.

## Supplemental Data

Supplemental Figure S1 Representative RPE/choroid flat mounts of *Cre*^*Trp1*^-based lines. Bright-field images of adult choroid/RPE flat mounts from four *Cre*^*Trp1*^-based lines; depigmented areas are indicated by **orange arrowheads** (peripheral RPE) and **red arrowheads** (central RPE). Scale bar = 1 mm.
