# Supplementary figures and images for: Role of HIF1α and HIF2α in Cre Recombinase–Induced Retinal Pigment Epithelium Pathology and Its Secondary Effect on Choroidal Neovascularization
Source: Am J Pathol. 2023 Jun 16;193(11):1694–705. doi: 10.1016/j.ajpath.2023.05.017 (PMC12178331; doi:10.1016/j.ajpath.2023.05.017)

Representative RPE/choroid flatmounts from *Cre<sup>Trp1</sup>* – based animals (adults)

Pigment (RPE)

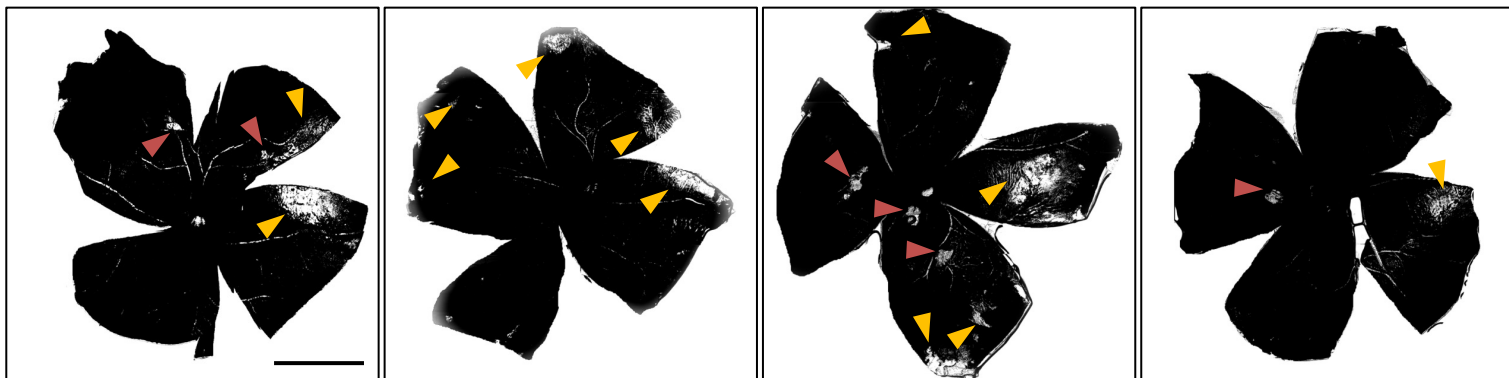

Supplement: Supplemental Figure S1 — Representative RPE/choroid flat mounts of CreTrp1-based lines. Bright-field images of adult choroid/RPE flat mounts from four CreTrp1-based lines; depigmented areas are indicated by orange arrowheads (peripheral RPE) and red arrowheads (central RPE). Scale bar = 1 mm. [file mmc1.pdf]
